# Molecular Detection and Characterization of *Rickettsia* Species in *Ixodid* Ticks Collected From Cattle in Southern Zambia

**DOI:** 10.3389/fvets.2021.684487

**Published:** 2021-06-07

**Authors:** Simbarashe Chitanga, Kennedy Chibesa, Karen Sichibalo, Benjamin Mubemba, King S. Nalubamba, Walter Muleya, Katendi Changula, Edgar Simulundu

**Affiliations:** ^1^Department of Biomedical Sciences, School of Health Sciences, University of Zambia, Lusaka, Zambia; ^2^Department of Pathobiology, School of Veterinary Medicine, University of Namibia, Windhoek, Namibia; ^3^School of Life Sciences, College of Agriculture, Engineering and Sciences, University of KwaZulu-Natal, Durban, South Africa; ^4^Centre for Infectious Diseases Research in Zambia, Lusaka, Zambia; ^5^Department of Wildlife Sciences, School of Natural Resources, Copperbelt University, Kitwe, Zambia; ^6^Department of Biomedical Sciences, School of Medicine, Copperbelt University, Ndola, Zambia; ^7^Department of Clinical Studies, School of Veterinary Medicine, University of Zambia, Lusaka, Zambia; ^8^Department of Biomedical Sciences, School of Veterinary Medicine, University of Zambia, Lusaka, Zambia; ^9^Department of Paraclinical Studies, School of Veterinary Sciences, University of Zambia, Lusaka, Zambia; ^10^Department of Disease Control, School of Veterinary Medicine, University of Zambia, Lusaka, Zambia; ^11^Macha Research Trust, Choma, Zambia

**Keywords:** *Rickettsia*, ticks, tick-borne zoonoses, Zambia, febrile illness

## Abstract

Tick-borne zoonotic pathogens are increasingly becoming important across the world. In sub-Saharan Africa, tick-borne pathogens identified include viruses, bacteria and protozoa, with *Rickettsia* being the most frequently reported. This study was conducted to screen and identify *Rickettsia* species in ticks (Family *Ixodidae*) infesting livestock in selected districts of southern Zambia. A total of 236 ticks from three different genera (*Amblyomma, Hyalomma*, and *Rhipicephalus*) were collected over 14 months (May 2018–July 2019) and were subsequently screened for the presence of *Rickettsia* pathogens based on PCR amplification targeting the outer membrane protein B (*ompB*). An overall *Rickettsia* prevalence of 18.6% (44/236) was recorded. Multi-locus sequencing and phylogenetic characterization based on the *ompB, ompA*, 16S *rRNA* and citrate synthase (*gltA*) genes revealed the presence of *Rickettsia africae* (*R. africae*), *R. aeschlimannii*-like species and unidentified *Rickettsia* species. While *R. aeschlimannii*-like species are being reported for the first time in Zambia, *R. africae* has been reported previously, with our results showing a wider distribution of the bacteria in the country. Our study reveals the potential risk of human infection by zoonotic *Rickettsia* species and highlights the need for increased awareness of these infections in Zambia's public health systems.

## Introduction

Vector–borne zoonotic pathogens are of increasing importance worldwide, with many reports of emerging and/or re-emerging pathogens being detected in invertebrate hosts (1). This increase in reports has been attributed to factors such as climate change, land-use changes as well as various anthropogenic activities (2). Amongst the emerging and re-emerging vector-borne pathogens, are those which are transmitted by ticks (3).

Ticks are considered to be amongst the main vectors of zoonotic pathogens (4), and rank only second to mosquitoes in terms of importance as vectors of human pathogens (5, 6). Ticks serve as reservoirs, vectors or amplifying hosts for a variety of pathogens, with a number of these ticks reportedly infesting humans (7–12). Tick-borne zoonoses are emerging across the world (13–15), with their public health impact being on the rise in tropical and subtropical regions (3, 16–18).

Within southern Africa, several human infections by tick-borne zoonotic pathogens have been reported. These include viral (Crimean-Congo hemorrhagic fever), bacterial (*Borrelia duttonii, Anaplasma phagocytophilum, Ehrlichia ruminantium, Ehrlichia canis, Rickettsia africae, Rickettsia aeschlimannii*, and *Rickettsia conorii*) and protozoal (*Babesia microti*) infections (19). The most commonly reported tick-borne pathogen in southern Africa is the obligate intracelluar bacteria of the *Rickettsia* genus (spotted fever group—SFG), which cause febrile illnesses in humans (20, 21). Across Africa, more than 10 SFG Rickettsiae have been reported in ticks, humans and animals (21). Despite these reports of the pathogens in Africa, the diseases they cause are still neglected (22).

Many human rickettsial infections have been reported within southern Africa, mostly in tourists. Countries within the region which have reported active human infections include South Africa (23–34), Botswana (35), Mozambique (36), and Zimbabwe (37, 38). Although there have been no confirmed clinical cases reported in Zambia, serological evidence of human infection exists (39). Furthermore, infection by rickettsial pathogens has been reported in non-human primates in the country (40). However, information on rickettsial pathogens in ticks is very limited. Presently, to our knowledge, there are only two published reports of *Rickettsia* in Zambian ticks: one from a tick survey (41), and the other from a tick collected from an exported reptile (42). Considering that ticks play a significant role in the epidemiology of these pathogens, it is essential that they are surveyed to assess the presence of pathogens, which can inform on the potential risk for human infections. Therefore, this study sought to screen and to phylogenetically characterize rickettsial pathogens from ticks collected from cattle in southern Zambia.

## Materials and Methods

### Tick Collection, Identification, and DNA Extraction

**Figure 1:**
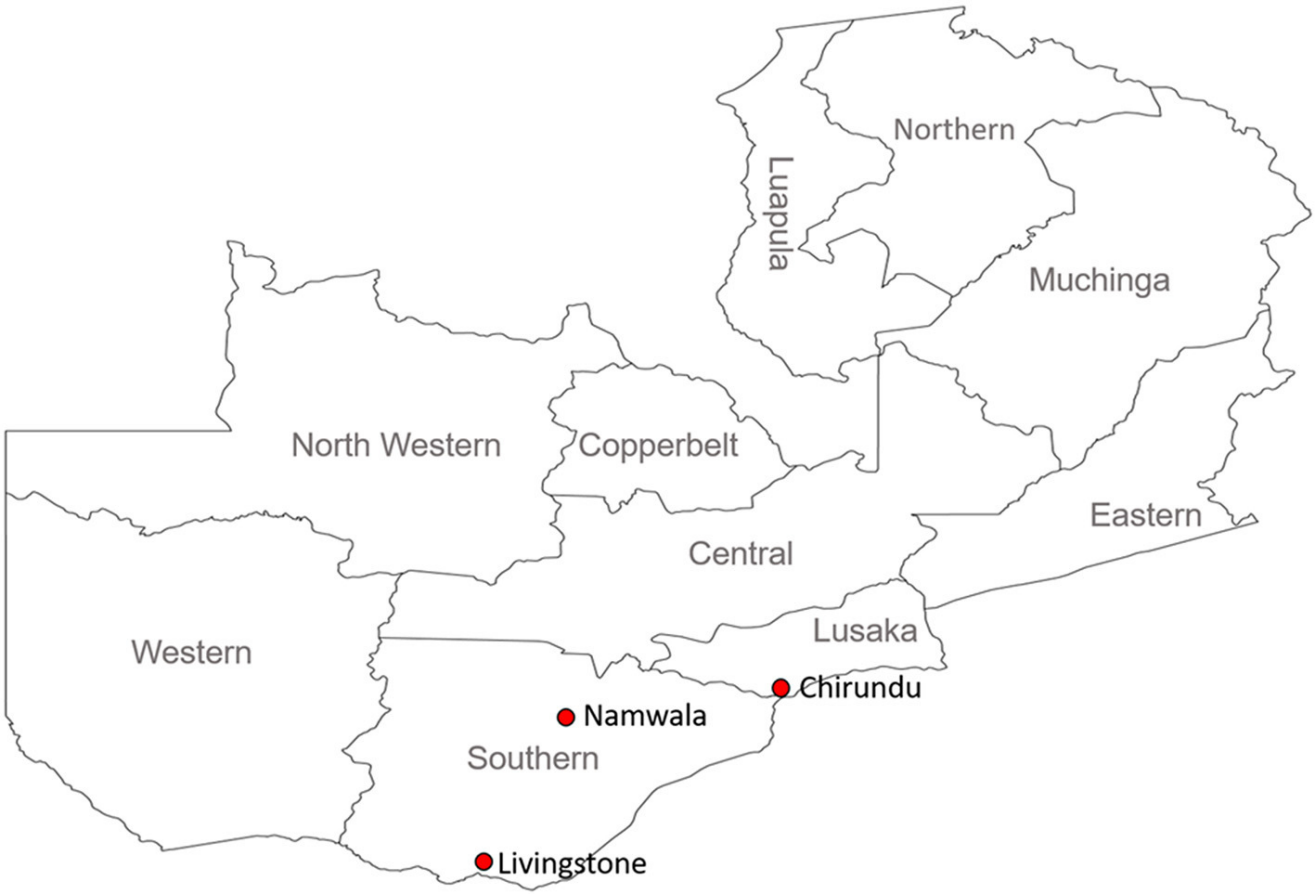
Map of Zambia showing the study sites (Red dots) in southern Zambia. Sampling sites covered Lusaka (Chirundu) and Southern Provinces (Namwala and Livingstone).

During May 2018 to July 2019, Ixodid ticks were collected from cattle in three districts in the southern part of Zambia (Chirundu, Namwala, and Livingstone; Figure 1). The sampling areas were purposively chosen because the southern region has the highest livestock density in the country, with the majority of the people practising subsistence farming. There is therefore close interaction between the people and the animals. Cattle sampling was chosen as it widely reared and ensured easy collection of the tick samples. The ticks were placed in aerated tubes provided with moisture and transported live to the laboratory for morphological identification using identification keys (43). After morphological identification, the ticks were stored at −80°C awaiting further analysis. For molecular analysis, individual ticks were sterilized by washing briefly in 70% ethanol after which they were washed in phosphate buffered saline (PBS) and transferred into homogenizing tubes containing 200 μL of Dulbecco's modified eagle medium (DMEM) (Sigma–Aldrich®–USA). The ticks were then homogenized in a MicroSmash™ MS-100R homogenizer (TOMY Digital Biology Co., Ltd., Japan), after which the homogenate was centrifuged, with the supernatant separated into a clean micro-centrifuge tube. DNA was extracted from the tick homogenate using the TRI Reagent® protocol (Sigma–Aldrich, USA) according to the manufacturer's recommendation. The extracted DNA was used in subsequent polymerase chain reactions (PCR) to screen for the presence of rickettsial DNA as well as sequencing.

### Molecular Screening and Phylogenetic Analysis of *Rickettsia*

For initial PCR screening, OneTaq® Quick-Load® 2X Master Mix with Standard Buffer (New England BioLabs® Inc., USA) was used to amplify a 429-bp region of the outer membrane protein B (*ompB*), with cyclic conditions used previously (44). A negative control (using nuclease–free water in place of template DNA) was included in the PCR assays. The PCR primer pairs, expected amplicon size in base pairs (bp) and annealing temperatures of the assays are as shown in Supplementary Table 1. PCR products were electrophoresed on ethidium bromide stained 1.5% agarose gel and then visualized under ultraviolet (UV) light.

For sequencing, all samples positive on the *ompB* gene were purified using Wizard® SV Gel and Clean-Up System (Promega, Madison, WI, USA). Bidirectional Sanger sequencing was conducted with the purified DNA as a template using Brilliant Dye™ Terminator Cycle Sequencing Kit v3.1 (NimaGen®) according to the manufacturer's protocol. Nucleotide sequences were assembled and edited using GENETYX ATGC software version 7.5.1 (GENETYX Corporation, Tokyo, Japan). For phylogenetic analysis, reference sequences were retrieved from GenBank and aligned along with those determined in this study using ClustalX2. Phylogenetic trees were constructed in MEGA version 6.0 (45) using the Maximum Likelihood method based on the Tamura 3-parameter model and topological support was assessed using the bootstrap method with 1,000 replicates as a confidence interval.

Based on the results from phylogenetic analysis of the *ompB*, representative samples from each identified species were selected for amplification of a 532-bp fragment of the *ompA*, a 985-bp fragment of the 16S ribosomal ribonucleic acid (16S *rRNA*) and a 589-bp fragment of the citrate synthase (*glt*A) genes for further confimation of the species identity. The amplified segments were subsequently sequenced and phylogenetically analyzed as described for the *ompB* gene. All the primers used in this study are shown in Supplementary Table 1. The nucleotide sequences obtained in this study have been deposited in the GenBank with accession numbers LC565644–LC565678, LC565679–LC565695, LC565696–LC565701, and LC565702–LC565706 for *ompB, ompA, gltA*, and 16S rRNA genes, respectively.

## Results

### Tick Collection and Prevalence of Infection

A total of 236 engorged and semi-engorged adult ticks were collected (Chirundu-36, Namwala-100, and Livingstone-100). On morphological identification, these included *Amblyomma* spp. (19), *Hyalomma* spp. (99), and *Rhipicephalus* spp. (118). The distribution of tick genera in the study areas is shown in Table 1.

**Table 1:** Number of ticks collected and the prevalence of infection in the different sampling areas by genus.

**Tick genus**	**Number of ticks analyzed (Prevalence)**			**Total number of ticks (Prevalence)**
	**Chirundu**	**Namwala**	**Livingstone**	
*Amblyomma* sp.	1 (0%)	14 (28.6%)	4 (75%)	19 (36.8%)
*Hyalomma* sp.	31 (9.7%)	0 (0%)	68 (42.6%)	99 (31.3%)
*Rhipicephalus* sp.	4 (0%)	86 (5.8%)	28	118 (5.8%)
Total	36 (8.3%)	100 (9%)	100 (32%)	236 (18.6%)

### Molecular Detection of *Rickettsia*

Based on the initial screening targeting the *ompB* gene, the observed overall prevalence of *Rickettsia* in the ticks was 18.6% (44/236). All the sampled tick genera were found to harbor *Rickettsia* pathogens, with all sampling areas reporting a different prevalence of infection (Table 1).

### *Rickettsia* Species Identification

*Rickettsia* specific *ompB* nucleotide sequences obtained from all the 44 rickettsia-positive tick samples were compared to sequences in GenBank using the basic local alignment search tool (BLAST; https://blast.ncbi.nlm.nih.gov/Blast.cgi) (46). The sequences showed high similarity to *R. africae* (40.9%; 18/44), *R. aeschlimannii* (52.3%; 23/44), and *R. parkeri* (6.8%; 3/44), with sequence identity ranging from 98.3 to 99.77% (Supplementary Table 2).

On BLAST analysis of selected *ompA* sequences, there was an agreement between the *ompA* and *ompB* analysis on all samples which on the latter gene had shown close sequence similarity to *R. africae* (N383, N384, N385, N386, and N387), with 100% sequence similarity to *R. africae* KZN26 detected in South Africa (accession no. MH751466). The samples which had shown high sequence similarity to *R. parkeri* on *ompB* analysis (CT36, CT40, and CT43), showed 100% sequence similarity to *R. africae* which was found in India [accession no. MK905242]. Meanwhile, samples which had shown close sequence similarity to *R. aeschlimannii* on *ompB* analysis (N320, N323, N330, N345, N349, N356, N358, and N377), showed high sequence similarity (99.81–100%) to a *Rickettsia* endosymbiont from Turkey (accession no. KT279888), with the exception of sample N381 which showed 100% sequence similarity to *R. aeschlimannii* from Turkey [accession no. MG920562] (Supplementary Table 3).

BLAST analysis on *gltA* sequences showed agreement with the *ompB* sequence identity for *R. aeschlimannii* (N381), displaying 100% sequence similarity *R. aeschlimannii* from China [MH267736]. Meanwhile, CT36 which had shown high sequence similarity to *R. parkeri* on the *ompB* analysis, showed high sequence similarity to *R. africae* detected in Egypt [accession no. HQ335126] (99.87% identity) and those that had shown close sequence similarity to *R. africae* on *ompB* analysis (N28, N383, and N385), displayed 99.61% sequence similarity to *Rickettsia* sp. Identified in Slovakia [accession no. HM538186] (Table 2).

**Table 2:** *Rickettsia* identity based on the gltA gene.

**Area**	**Tick species**	**DNA ID**	**Reference strains of *Rickettsia* species (GenBank Accession Number)**	**Nucleotide percent identity (%)**
Chirundu	*Hyalomma* spp.	CT36	*R. africae* Egypt (HQ335126)	99.87
Namwala	*Amblyomma* spp.	N28	*Rickettsia* sp. Slovakia (HM538186)	99.74
Namwala	*Rhipicephalus* spp.	N61	*Rickettsia* sp. Slovakia (HM538186)	99.61
Livingstone	*Hyalomma* spp.	N381	*R. aeschlimannii* China (MH267736)	100
Livingstone	*Amblyomma* spp.	N383	*Rickettsia* sp. Slovakia (HM538186)	99.87
Livingstone	*Hyalomma* spp.	N385	*Rickettsia* sp. Slovakia (HM538186)	99.61

The *16S rRNA* gene BLAST sequence analysis results revealed agreement with the findings of *ompB* analysis for samples with close sequence similarity to *R. africae* (N61, N383) and *R. aeschlimannii* (N323, N381) with sequence identity ranging from 99.78 to 100% to those detected in Ethiopia [L36098] and China [MH923218], respectively. Meanwhile, the sample that had shown close sequence similarity to *R. parkeri* on *ompB* analysis (CT36), showed highest sequence similarity to an uncultured *Rickettsia* sp. Strain Bel-4109 (MH618379) from Serbia (Table 3).

**Table 3:** *Rickettsia* identity based on the 16S rRNA gene.

**Area**	**Tick species**	**DNA ID**	**Reference strains of *Rickettsia* species (GenBank accession number)**	**Nucleotide percent identity (%)**
Chirundu	*Hyalomma* spp.	CT36	*Rickettsia* sp. Strain Bel–4109 (MH618379)	100
Namwala	*Rhipicephalus* spp.	N61	*R. africae* Ethiopia (L36098)	100
Livingstone	Hyalomma spp.	N323	*R. aeschlimannii* China (MH923218)	100
Livingstone	Hyalomma spp.	N381	*R. aeschlimannii* China (MH923218)	100
Livingstone	Amblyomma spp.	N383	*R. africae* Ethiopia (L36098)	99.78

### Phylogenetic Analysis

**Figure 2:**
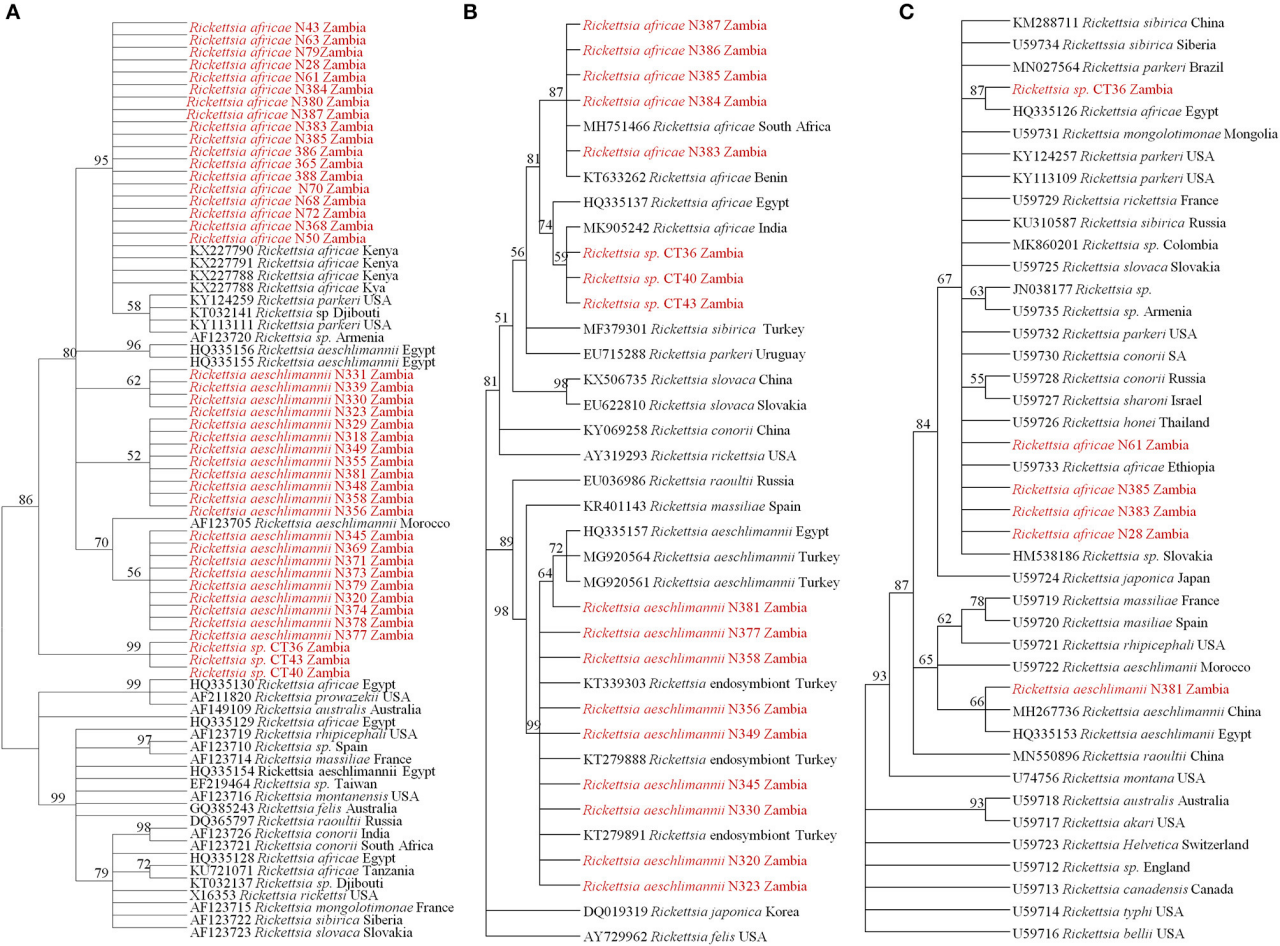
Phylogenetic relationships of the *ompB* **(A)**, *ompA* **(B)**, and *gltA* **(C)** genes of *Rickettsiae* detected from ticks in Zambia. Phylogenetic analysis was based on 397, 460, and 596 bp of the *ompB, ompA*, and *gltA* genes, respectively. Numbers at branch nodes indicate bootstrap values ≥50%. The reference sequences included in the analyses are shown by their GenBank accession number, species, and country of origin. The *Rickettsiae* characterized in the present study are in red text.

**Figure 3:**
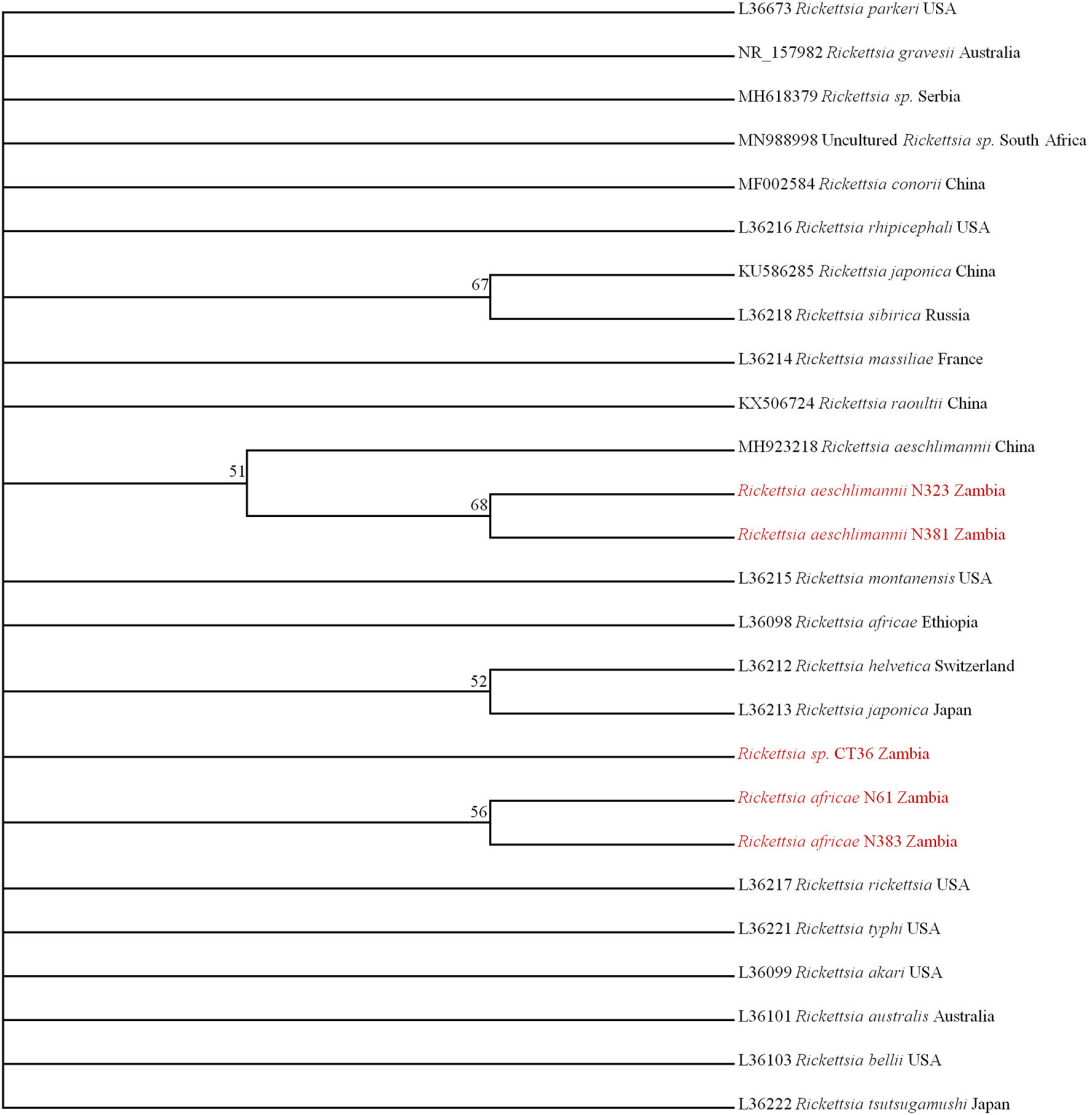
Phylogenetic relationships of the *16S rRNA* genes of rickettsiae detected in ticks in Zambia. Phylogenetic analysis was based on 795 bp of the 16S *rRNA* gene. Numbers at branch nodes indicate bootstrap values ≥50%. The reference sequences included in the analyses are shown by their GenBank accession number, species, and country of origin. The *Rickettsiae* characterized in the present study are in red text.

On phylogenetic analysis based on the *ompB* sequence, the Zambian sequences under study formed three distinct clusters, namely *R. africae, R. aeschlimannii*, and *Rickettsia* sp. (Figure 2A). It was noteworthy that the Zambian *Rickettsia* sp. did not cluster with any of the reference sequences from GenBank. Phylogenetic analysis based on the *ompA* sequence showed Zambian sequences forming two clusters; one grouping with *R. africae* and another clustering with *R. aeschlimannii* and *Rickettsia* endosymbiont (Figure 2B). In addition, phylogenetic analysis of selected samples, based on the *gltA* sequence showed that Zambian samples clustered with *R. africae* (CT36, N61, N385, N383, and N28) and *R. aeschlimannii* (N381) (Figure 2C). Based on 16S *rRNA* gene sequences, phylogenetic analysis of the Zambian samples clustered with *R. africae* (N61, N383), *R. aeschlimannii* (N323, N381) with CT36 not clustering with any reference sequences from GenBank (Figure 3).

Taken together, based on nucleotide sequence similarities as determined by BLAST analysis and phylogenetic analysis of four different genes, there were three distinct *Rickettsia* species identified, namely *R. africae, R. aeschlimannii*-like species and *Rickettsia* sp.

### Distribution of *Rickettsia* Species by Sampling Area and Tick Genera

By area of sampling, ticks from Chirundu were infected with *Rickettsia* sp. (3/3), those from Namwala were infected by *R. africae* only (9/9), whilst those from Livingstone were infected by *R. africae* (9/32) and *R. aeschlimannii*-like species (23/32)]. By tick genera, *Amblyomma* were infected with *R. africae* (6/7) and *R. aeschlimannii*-like species (1/7), *Hyalomma* were infected with *R. aeschlimannii*-like species (22/32), *R. africae* (7/32), and unidentified *Rickettsia* sp. (3/32), with *Rhipicephalus* ticks being found only with *R. africae* (5/5).

## Discussion

This study found an overall *Rickettsia* prevalence of 18.6% in the ticks sampled from the southern part of Zambia. The previous report (41) on *Rickettsia* prevalence in ticks in Zambia showed a much lower prevalence of 4.6% and the difference in prevalence between the two studies could be attributed to the differences in the proportions of ticks sampled. The study conducted in eastern Zambia by Chitimia-Dobler et al. (41) was predominated by ticks of the *Rhipicephalus* genus (96% *Rhipicephalus*, 4% *Amblyomma*, and *Hyalomma*), which are considered to always have lower rickettsial infections (41). In contrast, our study had relatively more *Amblyomma* and *Hyalomma* species (50% *Amblyomma* and *Hyalomma*, 50% *Rhipicephalus*), tick species considered amongst the principal *Rickettsia* vectors in the region (21). Indeed, our findings on infection rates in the three tick genera confirm the observations by Parola et al. (21) that *Amblyomma* and *Hyalomma* have the highest infection rates of *Rickettsia*, with *Rhipicephalus* being amongst those with low infection rates (21). However, since we collected ticks from cattle, our finding of *Rickettsia* pathogens in the tick species cannot be conclusively used as an indicator of their potential vector role. This is because the identified pathogens could have been picked from cattle during feeding.

In this study, we report for the first time the presence of *R. africae* and *R. aeschlimannii*-like species in Ixodid ticks from the southern part of Zambia. Whilst *R. africae* has been reported before in ticks from the eastern part of Zambia (41, 42), this study reports for the first time the presence of *R. aeschlimannii*-like species in the country, adding to the number of SFG Rickettsiae which are reported in Zambia. The previously reported SFG Rickettsiae include *R. africae, R. massiliae, R. conorii* and *R. felis* (40–42, 47). To our knowledge, Zambia becomes the third country in southern Africa to report the presence of *R. aeschlimannii*-like species, with previous reports being in South Africa (48) and Zimbabwe (49). We also report unidentified *Rickettsia* sp. which showed distinct phylogenetic clustering pattern based on the four genes analyzed in this study, and thus could not be assigned a specific species. This observation further emphasizes the recommendations by Fournier et al. (50) to use multiple genes to ensure proper speciation of *Rickettsia* species. Alternatively, speciation could be achieved by sequencing the entire full lengths of several diagnostic genes. For example, Moron et al. (51) sequenced the full length *ompB* gene (~6,000 bp) to phylogenetically compare *R. felis* with its homologs in spotted fever group (SFG) and typhus group (TG) *Rickettsiae*.

Our findings add to available data on the prevalence of rickettsial pathogens in ticks within the southern African region. Other countries in the region which have reports of tick surveys for *Rickettsia* infection include Zimbabwe (49), Botswana (52), Mozambique (53), and South Africa (54–58) with prevalence ranging from as low as 3% to as high as 77%. This study also adds to the growing data on tick-borne (41, 42, 59–61) and tick-associated (62, 63) zoonotic pathogens within Zambia. It further highlights the potential threat posed by tick-borne pathogens to human health and the need to strengthen surveillance of tick-borne diseases in the country.

In conclusion, we have shown the presence of zoonotic SFG *Rickettsiae* in ticks from the southern part of Zambia, indicating the wider geographical spread of these pathogens within the country and the potential threat they pose to human health. It is therefore important to screen for these pathogens in patients reporting with febrile illnesses of unknown origin. We also report on the presence of *R. aeschlimannii*-like species in Zambia, an indication of the expanded geographical spread of this pathogen within the southern African region.

## Data Availability Statement

The datasets presented in this study can be found in online repositories. The names of the repository/repositories and accession number(s) can be found in the article/Supplementary Material.

## Ethics Statement

The animal study was reviewed and approved by ERES Converge (IRB - 00005948, FWA - 00011697), under reference number 2017-Jul-021. Written informed consent for participation was not obtained from the owners because verbal consent was sought for collection of ticks from the animals.

## Author Contributions

SC and ES conceived the study. SC, KChi, KCha, and ES developed the laboratory methodology. KChi and KS conducted most of the laboratory experiments. SC, KN, KCha, and ES supervised the work. SC, KCha, and ES wrote the original draft of the manuscript. SC acquired funding for the study. All authors were involved in data analysis, reviewing, and approving the final version of the manuscript.

## Conflict of Interest

The authors declare that the research was conducted in the absence of any commercial or financial relationships that could be construed as a potential conflict of interest.
