# Supplementary material for: Molecular Detection and Characterization of Rickettsia Species in Ixodid Ticks Collected From Cattle in Southern Zambia
Source: Front Vet Sci. 2021 Jun 7;8:684487. doi: 10.3389/fvets.2021.684487 (PMC8215536; doi:10.3389/fvets.2021.684487)
Supplement: Supplementary file 1 [file Table_1.DOCX]

| *Table S1: Primers used in the study, including target genes and expected sizes of amplified fragments.* | | | | |
| --- | --- | --- | --- | --- |
| Target gene | PCR method | Primer name & sequence | Expected Band size (bp) | References |
| Outer membrane protein A (*omp*A) | Semi-Nested | **Rr190.70p**:  5′-ATGGCGAATATTTCTCCAAAA-3′ | 532 | Oteo et al., 2006 |
|  |  | **Rr190.701n**:  5′-GTTCCGTTAATGGCAGCATCT-3′ |  |  |
|  |  | **Rr190.602n**:  5′-AGTGCAGCATTCGCTCCCCCT-3′ |  |  |
| Outer membrane protein B (ompB) | Nested | ***ompB*-OF**:  5′-GTAACCGGAAGTAATCGTTTCGTAA-3′ | 429 | Choi et al., 2005 |
|  |  | ***ompB*-OR**:  5′-CTTTATAACCAGCTAAACCACC-3′ |  |  |
|  |  | ***ompB* SFG-IF**:  5′-GTTTAATACGTGCTGCTAACCAA-3′ |  |  |
|  |  | ***ompB* SFG/TG-IR**:  5′-GGTTTGGCCCATATACCATAA-G-3′ |  |  |
| 16S rRNA |  | **rrs-F21_342-362:**  5’-CCTGATCCAGCAATACCGAGT-3’ | 985 | This study |
|  |  | **rrs-R7_1306-1326:**  5’-CAAGGCCCGAGAACGTATTCA-3’ |  |  |
| Citrate synthase (*gltA*) | Semi – nested | **CS-F5_182-203:**  5’-GTGATAAAGGAATCTTGCGGCA-3’ | 589 | This study |
|  |  | **CS-R22_751-771:**  5’-TGAGGCAATACCCGTGCTAAT-3’ |  |  |
|  |  | **CS-R10_1018-1040:**  5’-GCGATAGCTTCAAGTTCTACTGC-3’ |  |  |
