# Supplementary material for: Molecular Detection and Characterization of Rickettsia Species in Ixodid Ticks Collected From Cattle in Southern Zambia
Source: Front Vet Sci. 2021 Jun 7;8:684487. doi: 10.3389/fvets.2021.684487 (PMC8215536; doi:10.3389/fvets.2021.684487)
Supplement: Supplementary file 2 [file Table_2.DOCX]

| *Table S2: Rickettsia identity based on the ompB gene* | | | | |
| --- | --- | --- | --- | --- |
| Area | **Tick species** | **DNA ID** | **Reference Strains of Rickettsia species (GenBank Accession Number)** | **Nucleotide percent identity (%)** |
| Chirundu | *Hyalomma spp.* | CT36 | *R. parkeri* USA (KY113111) | 99.02 |
| Chirundu | *Hyalomma* spp*.* | CT40 | *R. parkeri* USA (KY113111) | 99.02 |
| Chirundu | *Hyalomma* spp*.* | CT43 | *R. parkeri* USA (KY113111) | 99.02 |
| Namwala | *Amblyomma* spp. | N28 | *R. africae* Kenya (KX227790) | 99.53 |
| Namwala | *Amblyomma* spp. | N43 | *R. africae* Kenya (KX227790) | 99.53 |
| Namwala | *Rhipicephalus* spp. | N50 | *R. africae* Kenya (KX227791) | 99.30 |
| Namwala | *Rhipicephalus* spp. | N61 | *R. africae* Kenya (KX227790) | 99.29 |
| Namwala | *Amblyomma* spp. | N63 | *R. africae* Kenya (KX227790) | 99.53 |
| Namwala | *Rhipicephalus* spp. | N68 | *R. africae* Kenya (KX227791) | 99.53 |
| Namwala | *Amblyomma* spp. | N70 | *R. africae* Kenya (KX227791) | 99.53 |
| Namwala | *Rhipicephalus* spp. | N72 | *R. africae* Kenya (KX227791) | 99.53 |
| Namwala | *Rhipicephalus* spp. | N79 | *R. africae* Kenya (KX227790) | 99.53 |
| Livingstone | *Hyalomma* spp. | N318 | *R. aeschlimannii* Algeria MK028342) | 98.83 |
| Livingstone | *Hyalomma* spp. | N320 | *R. aeschlimannii* Algeria (MK028342) | 99.30 |
| Livingstone | *Hyalomma* spp. | N323 | *R. aeschlimannii* Algeria (MK028342) | 99.27 |
| Livingstone | *Hyalomma* spp. | N329 | *R. aeschlimannii* Algeria (MK028342) | 98.83 |
| Livingstone | *Hyalomma* spp. | N330 | *R. aeschlimannii* Algeria (MK028342) | 99.28 |
| Livingstone | *Hyalomma* spp. | N331 | *R. aeschlimannii* Algeria (MK028342) | 98.80 |
| Livingstone | *Hyalomma* spp. | N339 | *R. aeschlimannii* Algeria (MK028342) | 98.83 |
| Livingstone | *Hyalomma* spp*.* | N343 | *R. aeschlimannii* Algeria (MK02832) | 99.30 |
| Livingstone | *Hyalomma* spp. | N344 | *R. aeschlimannii* Algeria (MK028342) | 98.83 |
| Livingstone | *Hyalomma* spp. | N345 | *R. aeschlimannii* Algeria (MK028342) | 99.76 |
| Livingstone | *Hyalomma* spp. | N348 | *R. aeschlimannii* Algeria (MK028342) | 99.28 |
| Livingstone | *Hyalomma* spp. | N349 | *R. aeschlimannii* Algeria (MK028342) | 98.81 |
| Livingstone | *Hyalomma* spp. | N355 | *R. aeschlimannii* Algeria (MK028342) | 98.83 |
| Livingstone | *Hyalomma* spp. | N356 | *R. aeschlimannii* Algeria (MK028342) | 99.28 |
| Livingstone | *Hyalomma* spp. | N358 | *R. aeschlimannii* Algeria (MK028342) | 99.28 |
| Livingstone | *Hyalomma* spp*.* | N365 | *R. africae* Kenya (KX227791) | 99.53 |
| Livingstone | *Hyalomma* spp. | N368 | *R. africae* Kenya (KX227791) | 99.56 |
| Livingstone | *Hyalomma* spp. | N369 | *R. aeschlimannii* Algeria (MK028342) | 99.30 |
| Livingstone | *Amblyomma* spp. | N371 | *R. aeschlimannii* Algeria (MK028342) | 99.30 |
| Livingstone | *Hyalomma* spp. | N373 | *R. aeschlimannii* Algeria (MK028342) | 99.30 |
| Livingstone | *Hyalomma* spp. | N374 | *R. aeschlimannii* Algeria (MK028342) | 99.30 |
| Livingstone | *Hyalomma* spp. | N377 | *R. aeschlimannii* Algeria (MK028342) | 99.30 |
| Livingstone | *Hyalomma* spp. | N378 | *R. aeschlimannii* Algeria (MK028342) | 99.30 |
| Livingstone | *Hyalomma* spp. | N379 | *R. aeschlimannii* Algeria (MK028342) | 99.30 |
| Livingstone | *Hyalomma* spp*.* | N380 | *R. africae* Kenya (KX227791) | 99.53 |
| Livingstone | *Hyalomma* spp. | N381 | *R. aeschlimannii* Algeria (MK028342) | 98.83 |
| Livingstone | *Amblyomma* spp. | N383 | *R. africae* Kenya (KX227791) | 99.30 |
| Livingstone | *Amblyomma* spp. | N384 | *R. africae* Kenya (KX227791) | 99.30 |
| Livingstone | *Hyalomma* spp. | N385 | *R. africae* Kenya (KX227791) | 99.30 |
| Livingstone | *Hyalomma* spp. | N386 | *R. africae* Kenya (KX227791) | 99.30 |
| Livingstone | *Hyalomma* spp. | N387 | *R. africae* Kenya (KX227791) | 99.30 |
| Livingstone | *Hyalomma* spp*.* | N388 | *R. africae* Kenya (KF660535) | 99.53 |
