# Supplementary material for: Molecular Detection and Characterization of Rickettsia Species in Ixodid Ticks Collected From Cattle in Southern Zambia
Source: Front Vet Sci. 2021 Jun 7;8:684487. doi: 10.3389/fvets.2021.684487 (PMC8215536; doi:10.3389/fvets.2021.684487)
Supplement: Supplementary file 3 [file Table_3.DOCX]

| *Table S3: Rickettsia identity based on the ompA gene* | | | | |
| --- | --- | --- | --- | --- |
| Area | **Tick species** | **DNA ID** | **Reference Strains of Rickettsia species (GenBank Accession Number)** | **Nucleotide percent identity (%)** |
| Chirundu | *Hyalomma* spp*.* | CT36 | *R. africae* India (MK905242) | 100 |
| Chirundu | *Hyalomma* spp*.* | CT40 | *R. africae* India (MK905242) | 100 |
| Chirundu | *Hyalomma* spp*.* | CT43 | *R. africae* India (MK905242) | 100 |
| Livingstone | *Hyalomma* spp. | N320 | *Rickettsia* endosymbiont Turkey (KT279888) | 100 |
| Livingstone | *Hyalomma* spp. | N323 | *Rickettsia* endosymbiont Turkey (KT279888) | 100 |
| Livingstone | *Hyalomma* spp. | N330 | *Rickettsia* endosymbiont Turkey (KT279888) | 100 |
| Livingstone | *Hyalomma* spp. | N345 | *Rickettsia* endosymbiont Turkey (KT279888) | 100 |
| Livingstone | *Hyalomma* spp. | N349 | *Rickettsia* endosymbiont Turkey (KT279888) | 100 |
| Livingstone | *Hyalomma* spp. | N356 | *Rickettsia* endosymbiont Turkey (KT279888) | 100 |
| Livingstone | *Hyalomma* spp. | N358 | *Rickettsia* endosymbiont Turkey (KT279888) | 100 |
| Livingstone | *Hyalomma* spp. | N377 | *Rickettsia* endosymbiont Turkey (KT279888) | 99.81 |
| Livingstone | *Hyalomma* spp. | N381 | *R. aeschlimannii* Turkey (MG920562) | 99.53 |
| Livingstone | *Amblyomma* spp. | N383 | *R. africae* South Africa (MH751466) | 100 |
| Livingstone | *Amblyomma* spp. | N384 | *R. africae* South Africa (MH751466) | 100 |
| Livingstone | *Hyalomma* spp. | N385 | *R. africae* South Africa (MH751466) | 100 |
| Livingstone | *Hyalomma* spp. | N386 | *R. africae* South Africa (MH751466) | 100 |
| Livingstone | *Hyalomma* spp. | N387 | *R. africae* South Africa (MH751466) | 100 |
